# Renal dysfunction in cardiovascular diseases and its consequences

**DOI:** 10.1007/s40620-020-00842-w

**Published:** 2020-09-01

**Authors:** Giacomo Deferrari, Adriano Cipriani, Edoardo La Porta

**Affiliations:** 1 Department of Cardionephrology, Istituto Clinico Ligure Di Alta Specialità (ICLAS), GVM Care and Research, Via Mario Puchoz 25, 16035 Rapallo, GE Italy; 2 Grown-Up Congentital Heart Disease Center (GUCH Center), Istituto Clinico Ligure Di Alta Specialità (ICLAS), GVM Care and Research, Rapallo, GE Italy; 3 Department of Internal Medicine (DiMi), University of Genoa, Genoa, Italy

**Keywords:** Cardiovascular disease, Heart failure, Venous congestion, Worsening renal function, Acute kidney injury, Chronic kidney disease

## Abstract

It is well known that the heart and kidney and their synergy is essential for hemodynamic homeostasis. Since the early XIX century it has been recognized that cardiovascular and renal diseases frequently coexist. In the nephrological field, while it is well accepted that renal diseases favor the occurrence of cardiovascular diseases, it is not always realized that cardiovascular diseases induce or aggravate renal dysfunctions, in this way further deteriorating cardiac function and creating a vicious circle. In the same clinical field, the role of venous congestion in the pathogenesis of renal dysfunction is at times overlooked. This review carefully quantifies the prevalence of chronic and acute kidney abnormalities in cardiovascular diseases, mainly heart failure, regardless of ejection fraction, and the consequences of renal abnormalities on both organs, making cardiovascular diseases a major risk factor for kidney diseases. In addition, with regard to pathophysiological aspects, we attempt to substantiate the major role of fluid overload and venous congestion, including renal venous hypertension, in the pathogenesis of acute and chronic renal dysfunction occurring in heart failure. Furthermore, we describe therapeutic principles to counteract the major pathophysiological abnormalities in heart failure complicated by renal dysfunction. Finally, we underline that the mild transient worsening of renal function after decongestive therapy is not usually associated with adverse prognosis. Accordingly, the coexistence of cardiovascular and renal diseases inevitably means mediating between preserving renal function and improving cardiac activity to reach a better outcome.

## Introduction

The heart and kidney are essential for cardiovascular (CV) homeostasis. Cardiac activity provides blood and oxygen to all the organs of the body, whereas the kidney plays a key role in the maintenance of fluid, electrolyte and acid–base equilibrium, in hemoglobin synthesis as well as in the clearance of metabolic waste products. Maintenance of hemodynamic homeostasis depends on many complex and delicate interactions between the heart and kidney [1]. This interaction is fine-tuned by neurohormonal activity, including renin–angiotensin–aldosterone system (RAAS), sympathetic nervous system (SNS) and atrial natriuretic peptides (ANP).

In the early 1800s, Richard Bright described for the first time the association between cardiac and kidney diseases [2], confirmed one century later [3]. Today, there is more awareness of the renal consequences of cardiovascular disorders (CVD) and vice versa, as well as of the accelerated progression of both organ failures influenced by the bidirectional heart-kidney interactions. The frequent coexistence of CV and kidney diseases has led to a proposal of cardiorenal syndromes (CRS) defined as “a complex pathophysiological disorder of the heart and the kidneys whereby acute or chronic dysfunction in one organ may induce acute or chronic dysfunction in the other organ” [4]. This classification provides a clinically oriented descriptive definition, however not yet tested in clinical practice or in clinical trials [5–7].

Even though renal dysfunctions in CVD are mainly the result of hemodynamic changes and neurohormonal activation, in clinical practice cardiorenal interactions are more complex for many reasons. In fact, among them the coexistence of CV and renal dysfunction often may be the result of shared traditional CV risk factors, such as hypertension, diabetes mellitus, obesity, lipid disorders and smoking, or of non-traditional CV risk factors such as inflammation, bone and mineral disorders, anemia and malnutrition [7–9].

In the nephrological field, while it is well known that when kidney diseases are the primary event, they favor the occurrence of CVD, it is less appreciated that when CVD are the initiating event they induce or aggravate renal dysfunctions that in turn are associated with further CV deterioration. In addition, in the clinical nephrology community the pathophysiology of renal dysfunctions in CVD is traditionally associated with reduced arterial renal perfusion and the role of renal congestion is at times overlooked.

In this review, renal dysfunctions in CVD, their renal and cardiac consequences and their related mechanisms will be discussed in the following dedicated sections.

## CVD and renal consequences

CV abnormalities, even subclinical ones, are frequently associated with a preexistent or de novo chronic kidney disease (CKD): i.e., estimated glomerular filtration rate (eGFR) < 60 ml/min and/or albuminuria or proteinuria, which can progress to end stage renal disease (ESRD) and favor CV morbidity and mortality (so-called chronic CRS). However, in acute CVD it is often difficult to discriminate the preexisting chronic renal abnormalities from the acute renal dysfunctions [10]. In fact, acute CVD are frequently associated also with acute worsening of renal function (WRF) or acute kidney injury (AKI) (so called acute CRS), even if their incidence is not rare in chronic CVD.

### Association of CVD with baseline CKD

CKD is found in 6–12% of the general population [11] but is up to at least five times more frequent in patients with CVD. The prevalence of CKD is a little lower in clinical trials that usually exclude more severe CKD (serum creatinine ≥ 2–3 mg/dl), so population-based studies provide more reliable data. In analyzed papers, glomerular filtration rate (GFR) is estimated by Cockroft and Gault, MDRD or CKD-EPI formulae [12–14].

The prevalence of baseline GFR < 60 ml/min has been reported in around 40–60% of patients with chronic heart failure (CHF) with both preserved or reduced ejection fraction [15–23], and in about 30–40% of patients with stable coronary artery disease (CAD), cerebrovascular or peripheral artery disease (PAD) [24, 25]. In these patients CKD usually precedes or coincides with the “onset” of heart failure (HF). Results of individual studies on the so-called chronic CRS are either meta-analyzed or reviewed [26–28].

The prevalence of GFR < 60 is similar or even higher in acute CVD, particularly in decompensated acute heart failure (AHF, 50–70%) [29–34], in acute coronary syndromes (ACS, 25–50%) [35–38], or in strokes (25–30%) [39, 40]. Again, the results of many studies are either meta-analyzed or reviewed [27, 28].

The prevalence of abnormal albuminuria or proteinuria is high in CHF (25–50%) [20, 41–44], and in ACS (15–20%) [45] relative to the general population (7%) [46]. Interestingly, even if in most patients albuminuria/proteinuria were associated with GFR < 60, many albuminuric or proteinuric patients had GFR ≥ 60, thus increasing the dimension of CKD [20, 42, 44, 47].

### Progression of preexistent CKD or de novo CKD in CVD

In patients with baseline CKD, the presence of stable CVD or subclinical CV abnormalities [left ventricular hypertrophy (LVH), augmented intima-media thickness or aortic calcifications] was associated with a more rapid progression of CKD, leading even to ESRD [48–53].

**Fig. 1:**
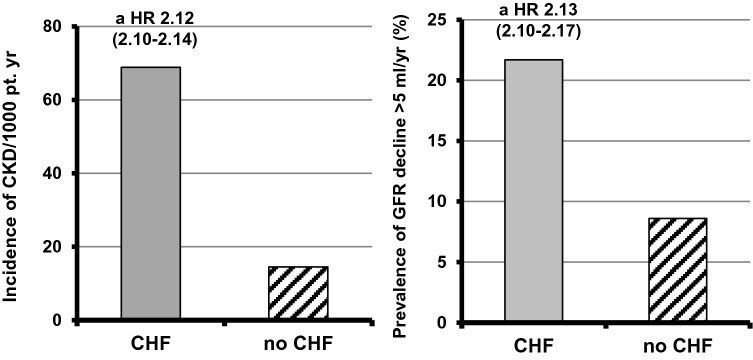
Incidence of CKD or GFR decline > 5 ml/min/year in patients with chronic heart failure (CHF) (156,743) or without CHF (3,414,122) (f up 3.6 year) (Drawn from data by George LK et al. Circ Heart Fail 2017 [54])

Elsayed and colleagues [48] firstly demonstrated that stable CVD are also independently associated with increased development of new CKD, an observation that was recently confirmed in CHF in a very large cohort of patients with normal basal GFR [54] (Fig. 1). Also subclinical abnormalities of the heart (i.e., LVH) or PAD were significantly associated with a faster decline in eGFR with the occurrence of de novo CKD [50, 54–57].

### Increased CV morbidity and mortality by CKD in CVD

GFR < 60 and its decline are independently associated with new CV events, rehospitalization and short- and long-term mortality in CHF [15–23, 26, 28, 58] and in chronic CAD, cerebrovascular disease or PAD [24, 25]. Also in AHF, ACS or acute stroke, GFR < 60 is significantly and independently associated with rehospitalization and short- and long-term mortality [29–33, 35–37, 39, 40]. The same was true for albuminuria or proteinuria in CHF [20, 41–44, 47] or ACS [45] even after adjusting for GFR. In summary, in CVD, CKD is frequently the most powerful predictor of morbidity and mortality, particularly when also the ratio of blood urea nitrogen to creatinine is higher than the normal range [59, 60].

### Incidence of WRF or AKI in CVD and their consequences

In CVD, in addition to baseline CKD, WRF or AKI are frequently observed mainly in hospitalized patients. WRF is arbitrarily defined as acute serum creatinine increased by ≥ 0.5 mg/dl or alternatively by ≥ 0.3 mg/dl sometimes associated with a creatinine increase by ≥ 25% [6, 61–66]. AKI is defined according to RIFLE, AKIN or KDIGO criteria [67–69]. In AHF, the use of different criteria seems to provide similar results in identifying acute renal dysfunction, its severity and also its capacity in predicting mortality [70, 71].

These acute complications are reported in about 15% of hospitalized patients with CHF [18, 28, 72] and more frequently in AHF (10–50%) [28, 34, 71, 73–79]. WRF/AKI are also reported in ACS (10–20%) [35, 37, 38, 63, 70, 80]. About 1/3–2/3 of acute renal dysfunctions are transient [10, 34, 76, 77, 80–83].

**Fig. 2:**
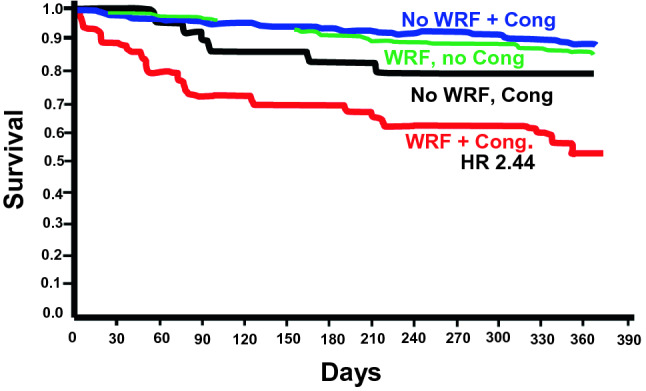
One-year death or urgent heart transplantation in acute heart failure (AHF) (594 patients) on the basis of worsening renal function (WRF) and signs of congestion at discharge (Adapted from Metra M et al. Circ Heart Fail 2012 [73])

In patients with CVD, not only baseline CKD, but also acute renal events are independently associated with rehospitalization and short- and long-term mortality in the above reported studies. Nevertheless, not all increases in serum creatinine have the same meaning and prognosis. In fact, in persistent WRF/AKI, the greater the severity of renal dysfunction, the greater the increase in mortality [10, 34, 35, 78, 81–83], even though in recent studies persistent WRF was significantly associated with mortality only in patients with residual congestion [73, 76, 78] (Fig. 2). Transient WRF was also associated with mortality, though this outcome occurred less often than in patients with persistent WRF [34, 35, 77, 81, 82]. In other studies, transient WRF was not significantly associated with increased mortality [10, 76, 83] particularly when mild [35].

Interestingly, a transient, mild WRF/AKI after patient decongestion may reflect adequate treatment and not necessarily a worsening of prognosis [73–76, 78, 84, 85] as frequently happens for WRF early after the initiation of RAAS inhibitors [86, 87]. Notably, one study found that even mild WRF/AKI were associated with long-term ESRD [63].

## Pathophysiology of renal dysfunctions in CVD and their consequences

### CKD in subclinical CVD or coronary, cerebrovascular and peripheral artery disease without heart failure (HF)

**Fig. 3:**
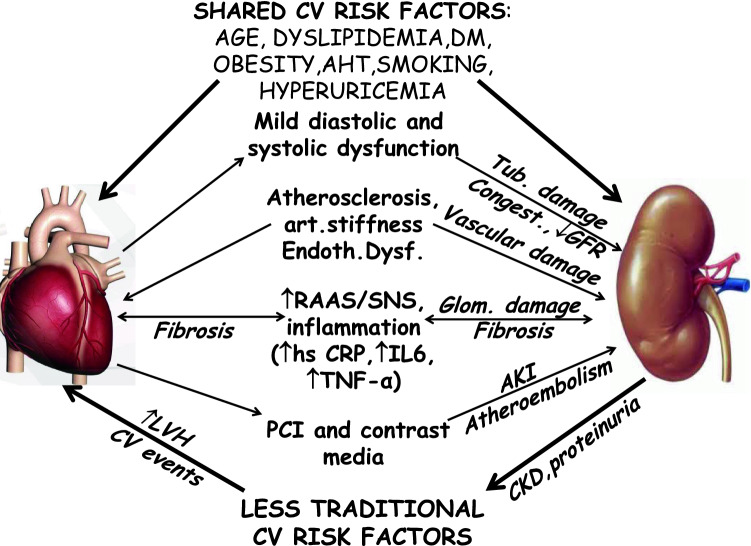
Pathophysiology of cardiorenal syndrome in subclinical cardiovascular disorders (CVD) or coronary, cerebrovascular and peripheral artery diseases without heart failure (HF)

Mechanisms of de novo CKD in these patients are poorly understood (Fig. 3). In long-term LVH (particularly if concentric) or stable CVD, shared traditional CV risk factors are frequently present [8, 48].

In addition, endothelial dysfunction, arterial stiffness and arteriosclerosis may affect renal vasculature [48, 49, 55, 88, 89]. In these patients, hemodynamic mechanisms, even an initial mild systolic and diastolic dysfunction, could have a consistent role [48, 50, 90, 91]. They can determine mild renal hypoperfusion and congestion with consequent subclinical inflammation and neurohormonal activation with tubular damage, glomerular (and also cardiac) fibrosis and proteinuria [50, 55]. An additional contribution could derive from conventional drug toxicity, and/or from occasional percutaneous interventions and contrast media [35, 36, 38, 48, 81]. Once CKD has been established, less traditional (CKD dependent) CV risk factors (Table 1) and the underuse of cardioprotective drugs and procedures can have an important role in worsening heart function [7, 9, 36, 38].

**Table 1:** Less traditional cardiovascular (CV) risk factors more frequent in chronic kidney disease (CKD)

Hyperactivation of RAAS
Sympathetic over-reactivity
Insufficient pressure-natriuresis (with consequent volume overload, arterial hypertension, venous congestion and heart failure)
CKD-related mineral and bone disorders( CKD-MBD): ↑ P, ↑ FGF23,↓ Kloto, ↑ PTH, ↑ propensity for vascular calcifications, Vitamin D deficiency
Endothelial dysfunction and nitric oxide inhibition
Atherosclerosis, intima media thickness, arterial stiffness
Inflammation (↑ CRP,↑ TNF-α, ↑ fibrinogen, ↑ Cytokines) and malnutrition
Accumulation of uremic toxins (ADMA, p-cresyl sulfate, indoxyl-sulfate, indole-3 acetic acid, trimethylamine N-oxide, etc.)
Hyperhomocysteinemia
Anemia (↓ EPO, iron depletion)
↑ Uric acid levels
Low or extremely high bicarbonate levels
Uremic dyslipidemia

*ADMA* asymetric dimethylarginine, *CRP* C-reactive protein, *FGF23* fibroblast growth factor 23, *EPO* erythropoietin, *PTH* parathyroid hormone, *RAAS* renin–angiotensin–aldosterone system, *TNF-α* tumor necrosis factor-α

### Renal dysfunctions in HF

**Fig. 4:**
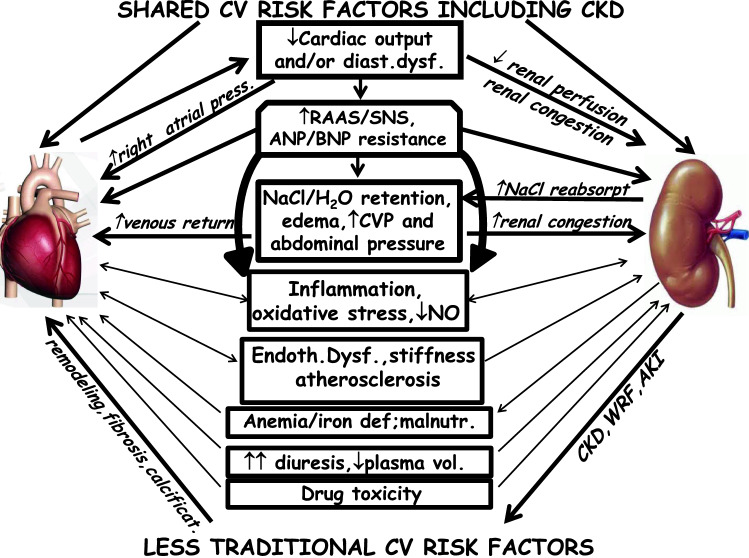
Pathophysiology of cardiorenal syndrome in heart failure (HF)

In HF the pathophysiology of renal dysfunction is complicated and multifactorial [6, 9, 66, 92–102] (Fig. 4).

Six categories of factors mainly contribute to renal and also cardiac outcomes in HF:

- shared traditional CV and renal risk factors;
- hemodynamic abnormalities due to systolic and/or diastolic dysfunction and congestion;
- impaired atrial contribution to diastolic ventricular filling in the case of atrial fibrillation
- SNS activation and the triggering of the RAAS and vasopressin;
- other factors such as inflammation, atherosclerosis, arterial stiffness and endothelial dysfunction, anemia ± iron deficiency, malnutrition, drug and procedure toxicity, in particular diuretic excess, and underuse of cardioprotective drugs;
- less traditional CV risk factors associated with CKD, including low GFR, (Table 1) and with vascular and valvular calcifications further worsening the heart condition.

GFR is determined by the pressure gradient between glomerular capillaries and the Bowman space according to the formula: GFR = K_f_[P_gc_ − P_bc_] − [π_gc_ − π_bc_] where K_f_ = filtration constant, P_gc_ = capillary hydrostatic pressure, P_bc_ = Bowman hydrostatic pressure, π_gc_ = capillary oncotic pressure and π_bc_ = Bowman oncotic pressure. According to this relationship, GFR is commonly reduced when P_gc_ is reduced (hypotension, low renal perfusion) and/or P_bc_ is increased (ureteral obstruction, renal congestion) [103, 104].

**Fig. 5:**
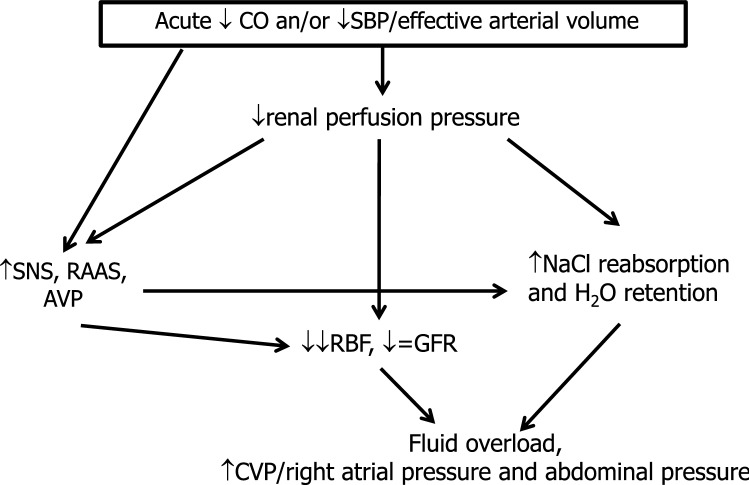
Impact of acute reduction in cardiac output (CO) and/or in systolic blood pressure (SBP)/effective arterial volume on renal function in heart failure (HF) (forward mechanism). *AVP* arginine vasopressin, *CVP* central venous pressure, *GFR* glomerular filtration rate, *RAAS* renin–angiotensin–aldosterone system, *RBF* renal blood flow, *SNS* sympathetic nervous system

According to the “low flow” or “forward failure” theory, in patients with HF with severe reduction of cardiac output, particularly when systolic blood pressure (SBP)/ effective arterial volume are reduced, renal perfusion pressure and renal blood flow (RBF) are reduced as well as GFR. SNS, RAAS, non-osmotic vasopressin and NO depletion are the most important mediators of intrarenal mechanisms of adaptation (Fig. 5) [6, 9, 92, 94–96, 105–109].

Interestingly, in mild reduction of cardiac output, GFR is maintained at an almost constant rate by an increased filtration fraction through intrinsic renal autoregulatory mechanisms such as afferent vasodilatation and predominant vasoconstriction of the efferent arteriolae with a secondary increase in postglomerular resistance. Both afferent vasodilatation and efferent vasoconstriction increase capillary hydrostatic pressure thereby counteracting the reduced renal perfusion. However, in severe reduction of cardiac output, vasoconstriction of also the afferent arteriolae ensues with an increase in preglomerular resistance, and the renal autoregulatory capacity is exhausted with a marked decrease in glomerular perfusion pressure and GFR. In this setting, non-hemodynamic factors such as inflammatory cytokine release, oxidative stress and endothelial dysfunction worsen the hemodynamic disorders and cooperate in further alterations of GFR.

The above-reported activation of neurohormonal axis directly and indirectly enhances also tubular reabsorption of NaCl and water, thus worsening fluid overload and congestion even in the presence of only mild reduction in cardiac output [108, 110–112]. Eventually, acute renal dysfunctions or even acute tubular necrosis could occur; tubulo-interstitial fibrosis and glomerulosclerosis resulting in worsening of renal function in CKD patients, leading to ESRD could be long-term consequences [94, 106, 113]. Thus, while the kidneys help to maintain homeostasis in healthy subjects, in HF they contribute to worsening CRS. Interestingly, similar responses are seen in HF with normal or increased cardiac output where neurohormonal adaptation, salt reabsorption and consequent blood volume expansion initially preserve renal perfusion [114].

Recent clinical data have shown that in persistent mild CHF or even in severe or acute cases, low cardiac output (“forward failure”) is not the major determinant of renal abnormalities but a great role is played by “backward failure”; this is particularly evident in right ventricular failure and/or in tricuspid regurgitation [115–118]. In fact, in HF, no correlation has been found between cardiac index and the reduction in GFR which is more closely associated with elevated central venous pressure or right atrial pressure even if their relationships are complex particularly in AHF [6, 96, 99, 100, 104, 119–128].

Interestingly, renal congestion detected by intraparenchymal Doppler venous pattern shows an independent and incremental role in predicting a worse outcome in CHF outpatients [129, 130] and perhaps WRF/AKI [131–133]. Moreover, it has been shown that backward failure impairs GFR preferentially in the presence of forward failure including low SBP [118, 119, 134–137].

Venous congestion in HF depends on fluid overload and/or cardiac dysfunction sometimes with the contribution of a transient decreased splanchnic capacity independent of fluid overload [109, 128, 138–140]. From half to two thirds of patients with AHF experienced clinical signs of congestion and/or no significant loss of weight during hospitalization and both were associated with significant adjusted increase in mortality [121, 123, 141–147] and in WRF [119, 121–123, 148].

Venous congestion affects renal veins where an increase in pressure ≥ 10–15 mmHg further alters glomerular hemodynamics, renal resistances, NaCl reabsorption and renin and inflammatory cytokine release [149–154]. Furthermore, in severe HF, the increased intra-abdominal pressure secondary to visceral edema and ascites further increases renal venous pressure as well as neurohormonal activation with consequent additional deterioration of GFR and sodium and water excretion [6, 94, 125, 128, 150, 155–159].

In fact, it was already demonstrated that humans with CHF have renal venous hypertension [151]: renal vein pressure was about 25 cm H_2_O (15–33) versus control values of 15 cm H_2_O (0.8–18). In those patients afferent, efferent and total renal resistance were markedly increased according to constriction of glomerular arteriolae, and both RBF and GFR were substantially decreased [151]. Interestingly, Bradley et al. in those years showed that in healthy subjects the experimental increase of renal vein pressure from 3–8 mmHg to 14–22 mmHg decreases urine flow and both RBF and GFR [150].

In the same period, several studies in experimental animals, in which renal venous pressure was increased to values observed in Maxwell’s patients, confirmed the reduction in urine flow, NaCl excretion, RBF and also GFR after a substantial increase in renal venous pressure [149, 160, 161]. These data were subsequently confirmed in non oliguric animals [162–164]. Notably, results in acute experiments are confirmed in dogs with renal vein hypertension of 3–4 weeks of duration [163]. In other experimental studies conducted in the same period it was shown that the increase in renal vein pressure similar to values observed in CHF, linearly increased interstitial, intratubular and Bowman hydrostatic pressure [153, 164–168], as well as renal vascular resistance in non denervated kidneys [169–171]. Similar experimental increases in renal venous pressure increased renin and aldosterone release [152, 156, 172, 173] as well as proteinuria [149, 173].

Recently, a reduction in RBF and GFR and an increase in interstitial hydrostatic pressure were observed in the congested kidney with a novel rat model of renal congestion [174]. Three days of renal congestion induced glomerular and tubular interstitial injury triggered by pericyte loss [174].

**Fig. 6:**
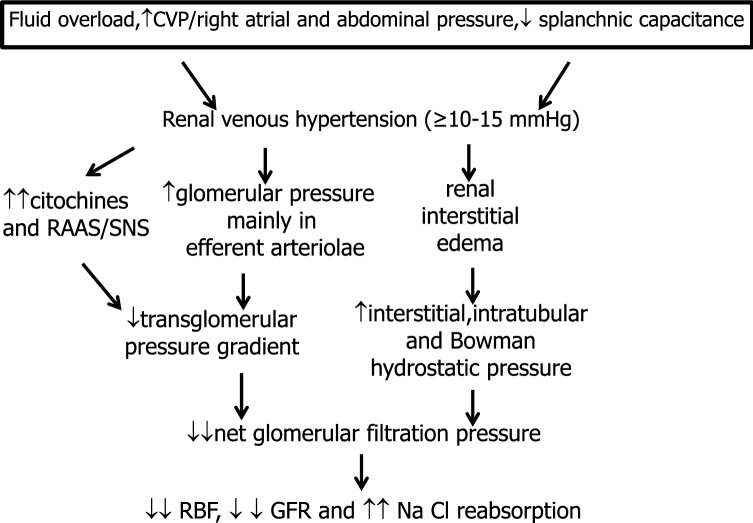
Impact of congestion on kidney function in heart failure (HF) (backward mechanism). *CVP* central venous pressure, *GFR* glomerular filtration rate, *RAAS* renin–angiotensin–aldosterone system, *RBF* renal blood flow, *SNS* sympathetic nervous system

In summary, in HF, forward and backward mechanisms frequently coexist and are strictly interconnected. The importance of congestion for explaining renal dysfunction is in part reported in many reviews [6, 93–96, 102, 104, 125, 127, 128, 139, 158, 159, 175] even though the mechanisms of the adverse effects of congestion on renal function are not fully elucidated. Clinical and physiological data reported in humans and also in experimental animals allow us to substantiate the concept that renal venous hypertension, together with SNS/RAAS activation, increases glomerular pressure in the efferent pole of glomerular capillaries (thus decreasing the A-V pressure gradient), and favors interstitial edema of the encapsulated kidney with an increase in interstitial, intratubular and Bowman hydrostatic pressure. As a consequence, the net filtration pressure is further reduced and consequently so is GFR; moreover, NaCl reabsorption is further increased and a vicious cycle is generated thereby worsening both cardiac and renal function (Figs. 5, 6). The renal effects of congestion are particularly evident in the presence of reduced cardiac output and/or SBP.

In these patients, even a mild reduction in cardiac output also increases the pressure in the right atrium, the ratio between right atrial pressure to pulmonary capillary wedge pressure and also increases the venous return owing to the fluid overload. Accordingly, the right cardiac filling pressure is further increased and the left ventricle is relatively underfilled (in diastole) with consequent further impairment of forward output [98, 118, 137, 176, 177].

As reported above (Table 1), also CV risk factors due to CKD or vascular or valvular calcification contribute to cardiac and renal damage and mortality [7–9].

### Acute renal dysfunctions (WRF/AKI) in CVD

In CVD, WRF/AKI, particularly if persistent, are markers of a severe setting in which both severe HF and CKD frequently coexist, which makes patients particularly vulnerable to acute renal dysfunctions [18, 35, 73, 81, 178–181]. So, all the mechanisms reported above to explain renal dysfunction are involved, in particular decreased renal perfusion, venous congestion with increased right atrial pressure and renal venous pressure, neurohormonal activation and inflammation. Other predisposing factors frequently detected by multivariable analysis are baseline CKD, diabetes, hypertension, vascular disease, old age and anemia [18, 35, 77, 79–81, 148, 178–180, 182, 183].

Among precipitating factors, the worsening of congestion, too little fluid loss or vice versa diuretic excess, a substantial decrease in SBP, nephrotoxic agents or percutaneous interventions with contrast media have to be considered [10, 18, 73–77, 79, 84, 85, 179, 180, 183–186].

How can AKI/WRF generate long-term mortality? First, they may be markers of CVD severity [18, 73, 181]. Second, when persistent, they may worsen CVD through fluid overload, anemia, neurohormonal activation and inflammation [6, 60, 70, 154]. Third, they can favor long-term ESRD further worsening CVD [63, 187, 188].

### Therapeutic approaches for treatment of HF with renal dysfunctions

The goal of treatment is to counteract the major modifiable pathophysiological abnormalities. So, the main target is to fight against hemodynamic abnormalities and to preserve euvolemia, pressure homeostasis and renal function. Another important goal is to avoid the underuse of CV drugs and interventions in patients with moderate to severe CKD [189–191]. In addition, it is necessary to differentiate WRF/AKI due to aggressive decongestion that is frequently transitory and with benign prognosis, from persistent dysfunctions. Finally, these patients should be treated early by a team involving cardiologists and nephrologists [191].

### Treatment of fluid overload and congestion

**Fig. 7:**
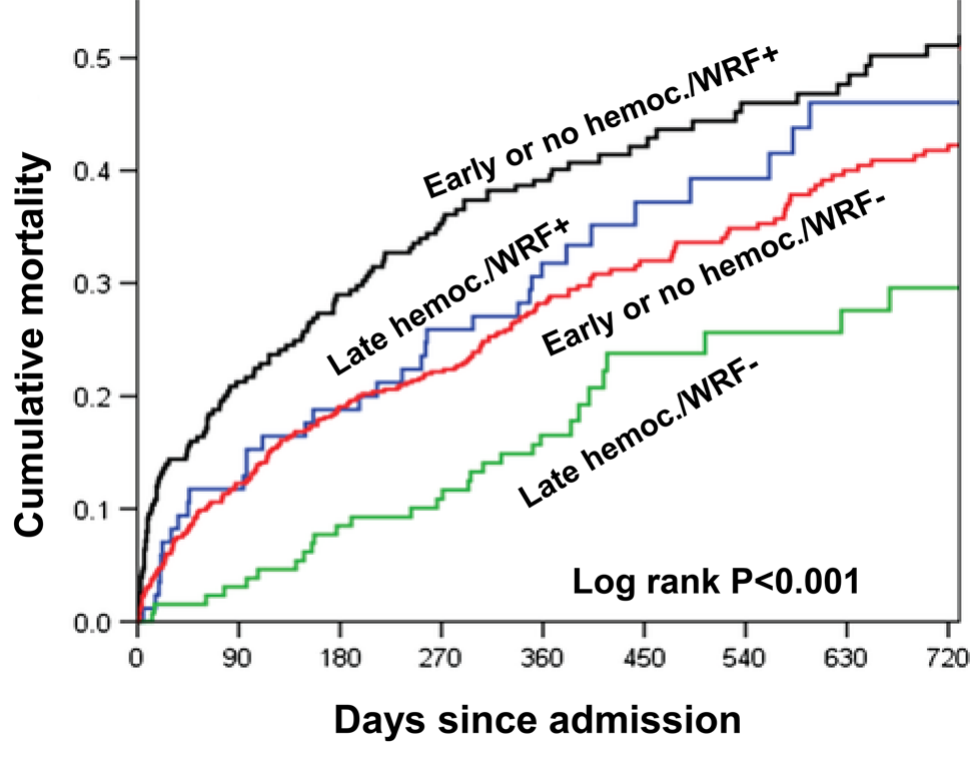
Mortality according to hemoconcentration and worsening renal function (WRF) in 1019 patients with acute heart failure (AHF) (Adapted from Breidthardt T et al. Eur J Heart Fail 2017 [75])

Ideally, congestion is prevented by initial salt (and water in hyponatremia) restriction [191, 192]. Diuretics are commonly used to treat fluid overload and renal congestion. Their dose must be tailored to not consistently exceed the interstitial mobilization of fluids to the vascular space [the so-called “plasma refill rate” (PRR)] which is continuously changing and in a relatively steady state condition is about 2.5–7 ml/min in hemodialyzed patients, varying with body size, capillary permeability, lymphatic flow, regional blood flow, serum protein levels and duration of decongestion [193–196]. A clinical surrogate of changes in PRR could be hemoconcentration regarding compounds (i.e., hemoglobin) or cells (i.e., red blood cells) confined in the intravascular compartment [194]: at a given moment, an increase in hematocrit or hemoglobin indicates that the removal of intravascular fluids exceeds PRR. In clinical practice, despite the evidence that hemoconcentration is associated with better outcomes, the removal of intravascular fluids consistently greater than PRR is unwise. In fact, the diuretic doses must be adequately tailored to avoid severe hypovolemia, hypotension, a further increase in RAAS activation, a further reduction of renal perfusion and GFR, and electrolyte disorders. Decongestion is associated with reduced mortality [74, 75, 84, 85, 197–199], and transient WRF after decongestion frequently has no negative impact on the prognosis [73–76, 78, 84, 85, 200]; indeed, late decongestion can offset the negative effects of WRF [75] (Fig. 7). Also the reduction in abdominal pressure was associated with a significant reduction in serum creatinine [157, 201].

Loop diuretics (furosemide or torasemide in furosemide-resistant patients) are the cornerstone of treatment. In severe CHF, the association of thiazide-like diuretics, such as metolazone and/or potassium-sparing diuretics (“sequential nephron blockade”) are often used to overcome the increased distal sodium reabsorption due to the chronic use of loop diuretics. In diuretic-resistant patients with metabolic alkalosis the association of acetazolamide to loop diuretics is particularly effective [190, 202].

Theoretically, congestion can also be reduced by increasing splanchnic vascular capacitance by ACE inhibitors (ACE-I) and/or β-blockers [140].

SGTL-2 inhibitors have recently shown important results in preventing hospitalization in diabetic patients with HF together with a reduction in the progression of renal disease. They have a diuretic effect (through a reduction of proximal sodium reabsorption and osmotic diuresis) and paradoxically a reduction of RAAS hyperactivity; in addition they inhibit cardiomyocyte Na/H exchanger and increase myocardial energetics [203–205]. Many trials were designed to evaluate the SGTL-2 effect in HF patients also without diabetes [203] and, interestingly, in November 2019 the DAPA-HF first demonstrated a reduction in CV mortality and HF hospitalization by SGTL-2 inhibitors in non-diabetic patients [203, 206].

In patients resistant to combined diuretic therapy, sodium and fluid retention were reduced by extracorporeal ultrafiltration with optimal results in most studies [207–209] also owing to the predictability of the amount of fluid removal and to the removal of cytokines and of isotonic fluids instead of hypotonic fluids which occurs with diuretics [97, 207, 208]. However, other trials obtained contrasting results [210, 211], also due to limitations in their design and conduction [212].

In the presence of severe congestion and stage IIIb, IV or V CKD, peritoneal dialysis could be a good therapeutic option to control both volume overload and uremic toxins and to enhance quality of life [213, 214].

### Neurohormonal blockers

International guidelines strongly recommend ACE-I or angiotensin receptor blockers (ARB) or mineralocorticoid-receptor antagonists (MRA) and β-blockers to improve survival and prevent hospitalization; recently, also angiotensin receptor-neprilysn inhibitors (ARNI) such as sacubitril-valsartan have been recommended [190–192]. In an updated network meta-analysis [215] in CHF better results on survival have been obtained by ACE-I plus MRA plus β-blockers or by ARNI plus MRA plus β-blockers. ACE inhibitors, ARB, MRA and ARNI improve renal perfusion and sodium and water retention counteracting the hemodynamic and neurohormonal imbalance and long-term cardiac and renal fibrosis that further deteriorate both cardiac and renal function [216]. These drugs initially worsen GFR (increase serum creatinine ~ 30%) particularly when SBP decreases to less than 80–90 mmHg; however, later changes in GFR over time could be lower than in controls as well as the risk of mortality [86, 87, 106]. These associations can be used also in the presence of CKD with special attention firstly to hyperkalemia in patients with less than 30 ml/min of GFR and secondly to maintain SBP not lower than 80–90 mm/Hg [190, 217]. They can also be used in patients with WRF/AKI without hemodynamic instability or hypotension, reducing the dose until renal function improves. Theoretically, neurohormonal blockers can have an additional favorable effect in congested patients augmenting splanchnic capacitance [109, 128, 138–140].

### Inotropic and vasopressor drugs

In refractory AHF patients with reduced ejection fraction and systolic blood pressure ≥ 90 mm/Hg, low dose dopamine, a renal vasodilator [218], could be useful to improve RBF and GFR even in the presence of WRF/AKI. In fact, dopamine infused at 1–5 μg/Kg/min with low/medium doses of diuretics maintains stable or increases GFR and reduces the incidence of WRF relative to high doses of diuretics ± dopamine [219–223]. Unfortunately, these data were overlooked in recent guidelines [190–192].

In refractory AHF patients with severe reduction of ejection fraction and with systolic blood pressure ≥ 85 mm/Hg, the infusion of levosimendan, an inotropic drug with arterial and venous dilatation properties, was associated with an improvement of GFR [224, 225], reduced mortality [226], and an increased risk of CV adverse events [192, 226]. Other inotropes seem associated with increased mortality [191, 192].

### Drugs counteracting nontraditional CV risk factors

Among them, in HF, the correction of anemia through intravenous iron improves NYAA class and symptoms, and reduces hospitalization [227]; a recent meta-analysis showed a significant reduction also in mortality [228]. Anemic patients with GFR less than 30–45 ml must be treated with erythropoietin, avoiding overtreatment at all times.

Promising results are also reported in chronic CRS with Cinacalcet that reduces FGS-23 levels [229, 230].

## Limitations

The present review has however some limitations. First, in CRS it is sometimes difficult: to understand the temporal causality of renal dysfunction; to highlight the role of traditional CV risk factors in simultaneously determining both cardiac and renal disorders; to distinguish their role from the direct contribution of CVD; and to discriminate the preexisting CKD from the acute renal dysfunction. In addition, studies are heterogeneous for many aspects such as selection bias, different inclusion criteria, formulae to estimate GFR and definition of acute renal dysfunction. Second, the effectiveness of diagnostic procedures to better predict risk is not completely understood. Third, mechanisms of adverse renal effects of congestion and the role of other factors such as inflammation, endothelial dysfunction and neurohormonal activation are not fully clarified. Moreover, renal congestion cannot be directly measured. Fourth, the ratio between long-term risks and benefits of therapeutic interventions, particularly on renal congestion, is not fully understood. Furthermore, data on PRR in CRS are lacking. Fifth, there is no strong clinical evidence of appropriate treatment. The few clinical trials that do exist are frequently retrospective, come from a single center and/or exclude severe renal dysfunction. Accordingly, large prospective trials are needed to better understand pathophysiological mechanisms and clinical results in CRS.

## Conclusions

In conclusion (Table 2), baseline CKD and AKI/WRF are frequently observed in patients with chronic and acute HF; both chronic and acute renal dysfunction are usually associated with a poor clinical outcome. CVD represent one of the most important causes of renal dysfunction. Renal congestion is a major contributing factor to renal dysfunction in HF. Finally, therapeutic principles for the treatment of CRS are described.

**Table 2:** Conclusions

1. In CVD:
Baseline CKD is up to 5 times more frequent than in the general population;
When CKD is present at baseline, the GFR decline is faster than in the general population, leading even to ESRD;
de novo CKD is frequently found;
CKD (GFR < 60 and/or albuminuria/proteinuria) is one of the most important factors independently predicting new CV events, rehospitalization, and mortality;
WRF/AKI occur in 50% of patients with AHF, slightly less frequently in CHF and ACS, and have a poor prognosis only if persistent and/or connected with residual congestion; transient WRF after decongestion may not result in a worse prognosis;
2. CVD may be considered one of the most important contributory causes of renal disease owing to the enormous diffusion of clinical and subclinical CVD and the high prevalence and incidence of renal abnormalities
3. Among the mechanisms of renal dysfunction in CVD, venous congestion is the most important; arterial renal hypoperfusion plays an important role particularly in acute severe reduction of cardiac output and/or of SBP
4. Therapeutic approaches for the treatment of HF with renal dysfunctions:
Treat fluid overload and congestion, and pay little attention to mild transient increases in serum creatinine after hemoconcentration;
Counteract hemodynamic and neurohormonal imbalance by RAAS inhibitors and/or β-blockers;
Infuse low dose dopamine in selected refractory AHF patients;
Counteract less traditional CV risk factors;
Avoid the underuse of CV drugs and interventions in CKD;
Early detect CRS for its effective management by a team involving cardiologists and nephrologists

*CVD* cardiovascular disease, *CKD* chronic kidney disease, *GFR* glomerular filtration rate, *ESRD* end stage renal disease, *WRF* worsening renal function, *AKI* acute kidney injury, *AHF* acute heart failure, *CHF* chronic heart failure, *ACS* acute coronary syndrome, *SBP* systolic blood pressure, *HF* heart failure, *RAAS* renin angiotensin aldosterone system, *CV* cardiovascular, *CRS* cardiorenal syndrome
